# The Effect of Do-It-Yourself Real-Time Continuous Glucose Monitoring on Glycemic Variables and Participant-Reported Outcomes in Adults With Type 1 Diabetes: A Randomized Crossover Trial

**DOI:** 10.1177/19322968231196562

**Published:** 2023-09-06

**Authors:** Shekhar Sehgal, Mona Elbalshy, Jonathan Williman, Barbara Galland, Hamish Crocket, Rosemary Hall, Ryan Paul, Robert Leikis, Martin de Bock, Benjamin J. Wheeler

**Affiliations:** 1 Department of Women’s and Children’s Health, Dunedin School of Medicine, University of Otago, Dunedin, New Zealand; 2 Department of Paediatrics, Canterbury District Health Board, Christchurch, New Zealand; 3 Te Huataki Waiora School of Health, The University of Waikato, Hamilton, New Zealand; 4 Te Whatu Ora, Capital, Coast and Hutt Valley, Wellington, New Zealand; 5 Te Whatu Ora, Hawke’s Bay, Hastings, New Zealand

**Keywords:** type 1 diabetes mellitus, continuous glucose monitoring, glycemic control, hypoglycemia, health-related quality of life, psychosocial outcomes

## Abstract

**Aim:**

Real-time continuous glucose monitoring (rtCGM) has several advantages over intermittently scanned continuous glucose monitoring (isCGM) but generally comes at a higher cost. Do-it-yourself rtCGM (DIY-rtCGM) potentially has benefits similar to those of rtCGM. This study compared outcomes in adults with type 1 diabetes using DIY-rtCGM versus isCGM.

**Methods:**

In this crossover trial, adults with type 1 diabetes were randomized to use isCGM or DIY-rtCGM for eight weeks before crossover to use the other device for eight weeks, after a four-week washout period where participants reverted back to isCGM. The primary endpoint was time in range (TIR; 3.9-10 mmol/L). Secondary endpoints included other glycemic control measures, psychosocial outcomes, and sleep quality.

**Results:**

Sixty participants were recruited, and 52 (87%) completed follow-up. Glucose outcomes were similar in the DIY-rtCGM and isCGM groups, including TIR (53.1% vs 51.3%; mean difference −1.7% *P* = .593), glycosylated hemoglobin (57.0 ± 17.8 vs 61.4 ± 12.2 mmol/L; *P* = .593), and time in hypoglycemia <3.9 mmol/L (3.9 ± 3.8% vs 3.8 ± 4.0%; *P* = .947). Hypoglycemia Fear Survey total score (1.17 ± 0.52 vs 0.97 ± 0.54; *P* = .02) and fear of hypoglycemia score (1.18 ± 0.64 vs 0.97 ± 0.45; *P* = .02) were significantly higher during DIY-rtCGM versus isCGM. Diabetes Treatment Satisfaction Questionnaire status (DTSQS) score was also higher with DIY-rtCGM versus isCGM (28.7 ± 5.8 vs 26.0 ± 5.8; *P* = .04), whereas diabetes-related quality of life was slightly lower (DAWN2 Impact of Diabetes score: 3.11 ± 0.4 vs 3.32 ± 0.51; *P* = .045); sleep quality did not differ between the two groups.

**Conclusion:**

Although the use of DIY-rtCGM did not improve glycemic outcomes compared with isCGM, it positively impacted several patient-reported psychosocial variables. DIY-rtCGM potentially provides an alternative, cost-effective rtCGM option.

## Introduction

Type 1 diabetes is a complex autoimmune disorder that requires the maintenance of glucose levels in a narrow target range.^1^ The majority of people with type 1 diabetes do not achieve glucose targets that are known to prevent long-term complications.^2^ However, continuous glucose monitoring (CGM) can improve diabetes outcomes and has overcome many of the limitations of capillary self-monitoring of blood glucose.^3^ Continuous glucose monitoring is now the recommended mode of glucose monitoring for type 1 diabetes in all major guidelines.^4,5^

There are two main types of CGM: intermittently scanned continuous glucose monitoring (isCGM) and real-time CGM (rtCGM). Although both improve outcomes in people with diabetes,^6^ rtCGM has several advantages over isCGM. Some of these have been partially addressed in second-generation isCGM devices,^7^ but ongoing advantages include the provision of continuously visible data, more flexible glucose threshold alerts, and remote monitoring. These have translated into improved glycemic and patient-reported outcomes for rtCGM versus first-generation isCGM in comparative studies.^8-10^ The six-month ALERTT1 study reported improved time in range (TIR), reduced hypoglycemia, increased achievement of glycemic control targets, and improved treatment satisfaction with rtCGM versus isCGM.^11^ However, cost is a key barrier to rtCGM, which has an annual cost of $3200 to $6400 compared with $1600 for isCGM,^12^ although both pricing and options for CGM are a rapidly evolving space.

**Figure 1.**
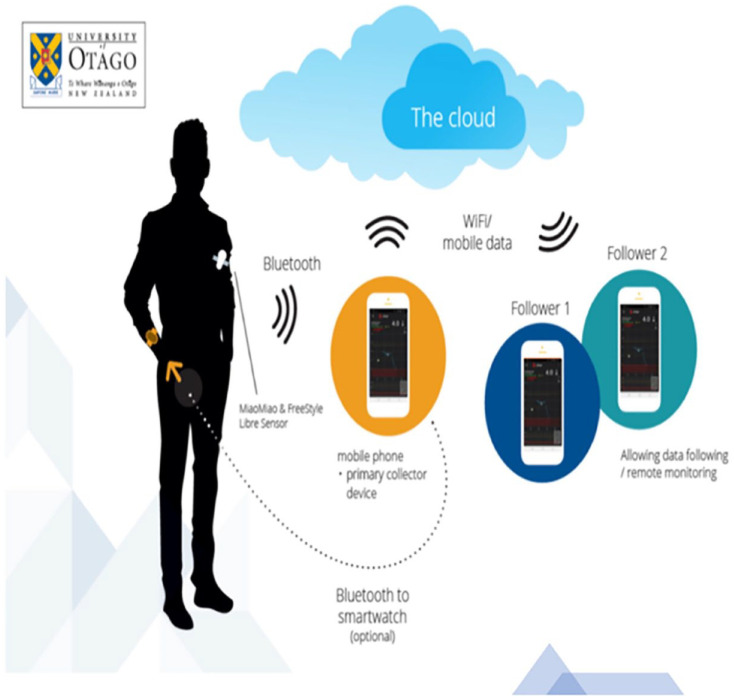
DIY-rtCGM device interconnectivity, including Fitbit Versa 2 smartwatch, first-generation FreeStyle Libre Sensor, MiaoMiao 2 Bluetooth transmitter, and Android phones with connections to Tidepool. The dotted line indicates mobile phone connectivity to smartwatch through Bluetooth, with the Wi-Fi symbols indicating Wi-Fi connectivity from phone to cloud (Tidepool) and phone to follower device. Abbreviation: DIY-rtCGM, do-it-yourself real-time continuous glucose monitoring.

Do-it-yourself (DIY)-rtCGM is a third-party rtCGM solution that has evolved from the #WeAreNotWaiting movement.^13^ When combined with isCGM and mobile phone–based software, many of the benefits of commercial rtCGM become available, including a full suite of user alarms and remote monitoring, at a cost of approximately $149.00 per rechargeable device.^12^ DIY-rtCGM uses near-field communication through a Bluetooth bridge to process raw isCGM sensor data and transmits this using Bluetooth to a paired smart device (Figure 1).^13^ A range of proprietary and open-source apps can be used to receive data and produce glucose readings. Smartwatch integration of glucose data is also possible. A recent randomized controlled crossover trial in children found modest improvements with DIY-rtCGM over isCGM for TIR and treatment satisfaction.^12^ No adult data are currently available.

Given that DIY-rtCGM has been adopted by many as a cheaper open-source rtCGM solution, more data are required on efficacy, psychosocial outcomes, and safety. Therefore, our objective was to explore differences between glycemic control and patient-reported outcomes between smart watch integrated DIY-rtCGM and first-generation isCGM in adults with type 1 diabetes.

## Methods

### Study Design

This multisite randomized controlled crossover study was conducted from May 2021 to November 2022 across six health regions in New Zealand (Southern, Waikato, Hawkes Bay, Capital and Coast, Taranaki, and Canterbury). The study protocol was approved by the Central Health and Disability Ethics Committee (21/CEN/74, Wellington, New Zealand). The study was registered with the Australian New Zealand Clinical Trials Registry (ACTRN 12621000648820) and issued a Universal Trial Number (U1111-1262–2784) by the World Health Organization International Clinical Trials Registry. Neither the isCGM nor the DIY-rtCGM manufacturers were involved in the planning, funding, or conduct of the study.

### Study Participants

Participants were informed about the study by their diabetes care provider during the course of routine clinical visits or through advertising on the Diabetes New Zealand web page. Inclusion criteria were age >16 years, type 1 diabetes duration of ≥6 months, and already using isCGM (only first-generation isCGM was available in New Zealand at study start). Exclusion criteria included pregnancy/planned pregnancy, use of oral diabetes medications or oral corticosteroids for >2 weeks prior to study start, severe diabetes-related complications (mild-moderate retinopathy and microalbuminuria were acceptable), current rtCGM use, or integrated sensor-augmented pump therapy, and severe medical or psychiatric comorbidities.

Full protocol details have been published.^14^ In brief, the study included an unblinded one-week run-in period to collect baseline glucose (through preexisting clinically used isCGM) and demographic data. Written informed consent was received from patients and/or their legal guardians as appropriate prior to commencing the trial.

### Randomization

Participants were randomly allocated to smartwatch-integrated DIY-rtCGM or to continue using first-generation isCGM (Free Style Libre 1; Abbott Diabetes Care Ltd., Witney, UK) for eight weeks. After a washout phase of four weeks, participants crossed over to use the other monitoring modality for a second eight-week phase. A 1:1 computer-generated randomization list, stratified by study site and glycemic control, was prepared by a statistician with no participant contact. The list was loaded onto the REDCap web application and group allocation was only revealed to participants and researchers at the completion of study visit 1.

### Participant Education and Quality Assurance

Participants were given a comprehensive education package provided over video conference by lead investigator S.S. Participants were followed up for any problem with their isCGM sensor and/or reader/collector. Each participant in both arms received standardized information over two visits.^6^ The educational content of these sessions followed the relevant principles from the European Association for the Study of Diabetes and the American Diabetes Association.^15^ The content of the education programs included adjustment of both bolus and basal insulin according to trend arrows/rate of change based on the work of Ajjan et al.^16^ All participants received appropriate training on the use of DIY-rtCGM and were provided with a user and troubleshooting guide.

In terms of quality assurance, participant uploads to Tidepool were a continuous process whenever the device was connected to Wi-Fi, with reviews happening every two weeks. Referring to the clinical consensus guidelines by Battelino, the uploads were analyzed for sensor quality as well as glucose information, aiming for >70% of sensor use over 14 days.^17^ If this was not achieved, potential barriers were discussed, and troubleshooting was provided through Zoom videoconference. This facility was potentially accessible at all times.

### Calibration

The system was calibrated by conducting capillary blood glucose, using a nationally funded CareSens N Pro meter (i SENS Seoul, Korea). The system can potentially be used without calibration, but this was recommended for additional safety, given the unknowns of DIY-rtCGM and had been used in a prior study.^12^ Participants were encouraged to calibrate once daily in the morning before breakfast when their glucose values were more likely to be stable although adherence to this aspect was not measured. Participants were reminded of the need to calibrate every study visit. Two glucose values taken on different fingers were utilized for calibration. Both calibration values were entered. When entered, the DIY-rtCGM sensor reading was immediately altered, in case of an incorrect calibration; once recognized, this could be replaced by a subsequent calibration as soon as the patient was able, thus reducing the potential bias related to incorrect calibration.

### Data Collection

During the DIY-rtCGM phase, participants wore a MiaoMiao 2 device attached over consecutive generation 1 Free Style Libre sensors. Glucose information was transmitted to an Android smartphone app (xDrip+) through Bluetooth. xDrip+ displayed standard CGM data and provided optional safety alerts for hypoglycemia and hyperglycemia. This app was also the primary source of communication with the Fit Bit Versa 2 smartwatch (Fit Bit Inc, San Francisco, CA, USA). The Glance open-source watch face (Ryan Mason 2019) was installed to conveniently view all DIY-rtCGM glucose data (Figure 1). To the best of our knowledge, there was no significant interference from other Bluetooth-enabled devices such as headphones because the Bluetooth link was mediated using Xdrip alone. Users could use Near Field Communication (NFC) to strengthen the DIY-rtCGM and phone connection. Users could use NFC to strengthen the DIY-rtCGM and phone connection. Alerts and Alarms were present on both the smartphone and smartwatch, and are able to be silenced on both devices individually. Participants were requested to respond to hypoglycemia/hyperglycemia alerts by conducting confirmatory capillary glucose testing.Recognizing the potential clinical consequences of data gaps in sensor glucose recordings, additional glucose testing was suggested in the first 24 hours and at any time that physical symptoms and sensor glucose did not match. Furthermore, if there was a sustained loss of connectivity for more than six hours, and which persisted following device reset, users were advised to disconnect their active DIY-rtCGM sensor and revert to capillary glucose testing and/or isCGM scanning while the study team arranged for technical support.

### Alarms and Alerts

At the initiation of DIY-rtCGM, three alert settings were recommended as per protocol.^14^ Users could also add predictive rise and fall of glucose alerts at their discretion. Alerts could be adapted as required, based on participant or clinician preference . The frequency of alert adjustment could not be measured independently in this study. Participants uploaded glycemic data to Tidepool (Palo Alto, CA USA) prior to the four study visits (weeks 1, 9, 13, and 21) and every two weeks during the study. During DIY-rtCGM, uploading was automatic using xDrip, and during isCGM this was completed manually. Participant glucose data were provided to the study team through the opensource app Tidepool (Palo Alto, CA, USA); information was shared with the diabetes care team and care partners with the participant’s consent. The participant’s own diabetes care team was responsible for adjusting insulin doses.

### Outcome Measures

The primary outcome was TIR, defined as the proportion of time with blood glucose at 3.9 to 10 mmol/L (70-180 mg/dL).^17^ The TIR was compared between groups during the final two weeks of each crossover phase. Standard secondary glycemic variables were also collected, in accordance with the international consensus on CGM trial reporting:^17^

- Time below range (<3.9 mmol/L/<70 mg/dL; including readings <3.0 mmol/L/<54 mg/dL)
- Time below range (<3.0 mmol/L/<54 mg/dL)
- Time above range (>10 mmol/L/>180 mg/dL; including >13.9 mmol/L/250 mg/dL)
- Time above range (>13.9 mmol/L/>250 mg/dL)
- Mean glucose ± standard deviation
- Coefficient of variation as an estimate of glucose variability
- Percentage of sensor data obtained by CGM devices (isCGM/DIY-rtCGM); sensor use.

Target control was defined as TIR ≥70%. Hypoglycemia was defined according to American Diabetes Association criteria as severe (level 3) with a capillary glucose of <3.0 mmol/L (54 mg/dL), requiring the assistance of another person or hospital admission due to impaired mental or physical functioning, or moderate (level 2) with a capillary glucose of <3.0 mmol/L (54 mg/dL) with symptoms of neurocognitive impairment that fall short of impaired function.^4^

### Participant-Reported Outcomes

Participants completed questionnaires to determine participant-related outcomes at weeks 1, 9, 13, and 21 in the order listed. These included the Diabetes Treatment Satisfaction Questionnaire (DTSQ) status and change, Gold Hypoglycemia awareness score, DAWN2 Impact of Diabetes profile (DIDP), Problem Area in Diabetes (PAID-20) questionnaire (participants ≥18 years) or PAID teen (participants <18 years), Hypoglycemia Fear Survey (HFS; behavior and worry subscales), Glucose Management Satisfaction Survey (GMSS), Pittsburgh Sleep Quality Index (PSQI), and the Diabetes Distress Survey (DDS)—Partner (see protocol paper for full details)^14^. In addition to this, a self-administered questionnaire on adverse events and hospitalization was also administered. A PAID score of ≥40 in adults was considered to indicate severe distress; in teenagers, the corresponding PAID-T score was ≥90 .^18,19^ Based on piloting within the study team, we estimated that 45 to 60 minutes were required to complete the surveys, which lead to zoom appointments of up to 60 minutes inclusive allocated to complete the surveys . Data on frequency of hospital admissions for diabetic ketoacidosis and severe hypoglycemia in the 12 months prior to study and during the study were also collected.

### Sample Size

Based on data from a pediatric study, it was estimated that 52 participants would provide 80% power to detect a 6% difference in TIR (SD = 15%) with a two-sided alpha of .05. The planned recruitment was 60 participants (30 per group) to allow for up to 15% attrition.

### Statistical Analysis

All analyses were performed based on original randomized group. Participants with missing study visits were excluded from final analysis. Differences in outcomes between the DIY-rtCGM and isCGM arms were determined using mixed linear regression models, with a random effect for participant and adjusted for period, sequence, and differences in baseline measures (at the start of each phase).^20^ Continuous data were described using mean and standard deviation. Categorical data were analyzed using proportions and McNemar’s test for paired binary data. Results were adjusted for multiple comparisons using the Benjamani–Hochberg approach.^21^ Stata 17 (Stata Corp, College Station, TX, USA) was used for statistical analysis. A two-sided *P* value <.05 was considered statistically significant. Result reporting was conducted according to the Consolidated Standards of Reporting Trials (CONSORT) 2010 statement extension for the reporting of crossover clinical trials.

## Results

### Study Population

**Figure 2.**
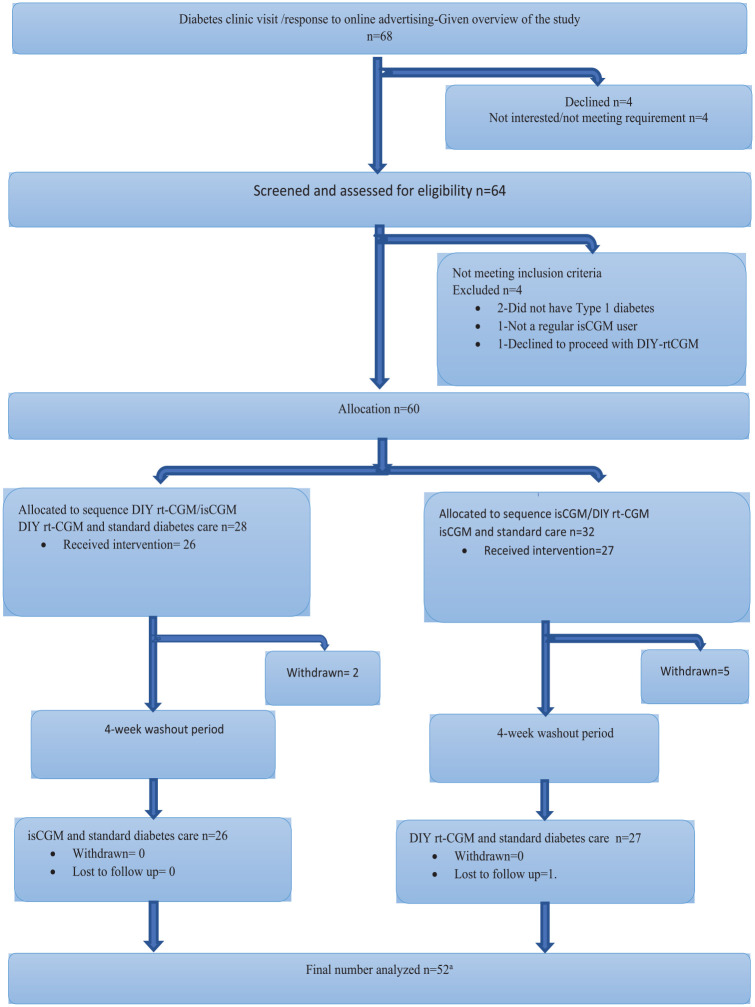
CONSORT diagram showing participant flow through the study. Abbreviations: CONSORT, Consolidated Standards of Reporting Trials; DIY-rtCGM, do-it-yourself real-time continuous glucose monitoring; isCGM, intermittently scanned continuous glucose monitoring. ^a^Study participants completing all four study visits.

A total of 60 participants were enrolled (mean age 37.6 ± 12.7 years, 59% women; Table 1). The trial recruited participants from May 2021 to June 2022, with follow-up completed in November 2022. Participant flow through the study is shown in Figure 2. Complete data were available for 52 participants (87%).

**Table 1.** Demographic and Clinical Characteristics of Study Participants at Baseline.

Characteristic	Participants (n = 60)
Age, years	37.6 ± 12.7
Female sex, n (%)	35 (59.3)
Body mass index, kg/m^2^	28.3 ± 0.2
Ethnicity, n (%)	
European	56 (93.3)
Māori^a^	3 (5.0)
Other	1 (1.7)
NZDep18^b^, n (%)	
Low deprivation (1-3)	19 (31.7)
Medium deprivation (4-7)	26 (43.3)
High deprivation (8-10)	15 (25.0)
Education, n (%)	
Some high school	2 (3.3)
High school	18 (30.0)
Tertiary	24 (40.7)
Postgraduate qualifications	16 (26.7)
Marital status, n (%)	
Married	42 (70.0)
Separated/divorced	2 (3.3)
Living alone	16 (26.7)
Insulin therapy, n (%)	
Multiple daily injections	31 (51.7)
Insulin pump	29 (48.3)

Values are given as mean ± SD or number of patients (%).

Abbreviation: NZDep, New Zealand Index of Deprivation

a Indigenous people of New Zealand.

b NZDep18 score is a deprivation index based on household location.

### Time in Range and Glycemic Outcomes

There was no evidence of a difference in TIR (primary outcome; *P* = .593; Table 2), or for any secondary glycemic metrics or glycosylated hemoglobin. There was also no difference between treatment arms in the proportion of participants achieving TIR ≥70% (23% vs 17%; *P* = .852, McNemar’s test).

**Table 2.** Glycemic Outcomes (n = 52).

	isCGM		DIY-rtCGM		Adjusted mean difference DIY-rtCGM vs isCGM [adjusted confidence intervals]^a^	*P* value
	Baseline (n = 52)	Eight weeks (n = 52)	Baseline (n = 52)	Eight weeks (n = 52)		
Time in range 3.9-10 mmol/L (70-180 mg/dL), %	53.2 ± 19.6	51.3 ± 15.4	54.2 ± 17.1	53.1 ± 17.4	−1.70 [−5.65, 2.25]	.593
Time at <3.9 mmol/L (<70 mg/dL), %	2.6 ± 2.8	3.8 ± 4.0	3.9 ± 4.0	3.9 ± 3.8	0.03 [−0.97, 1.03]	.947
Time at <3 mmol/L (<54 mg/dL), %	0.6 ± 1.5	0.9 ± 1.4	0.98 ± 1.8	1.52 ± 2.0	−0.55 [−1.4, 0.3]	.288
Time at >10 mmol/L (>180 mg/dL), %	43.5 ± 21.1	44.2 ± 17.0	42.1 ± 17.3	43.1 ± 18.1	1.10 [−4.53, 6.73]	.739
Time at >13.9 mmol/L (>250 mg/dL), %	17.1 ± 16.3	17.8 ± 2.1	14.8 ± 13.7	16.1 ± 14.1	2.10 [−3.10, 7.31]	.593
Mean glucose, mmol/L	9.96 ± 2.1	10.22 ± 2.1	10.9 ± 8.1	9.9 ± 1.8	0.95 [−1.62, 3.52]	.593
Coefficient of variance, %	35.5 ± 6.5	37.1 ± 6.3	37.0 ± 6.6	37.1 ± 9.4	0.68 [−2.2, 3.6]	.738
Sensors use, %	78.3 ± 28.0	84.4 ± 22.8	74.0 ± 30	73.8 ± 27.1	13.2 [−4.5, 30.9]	.200
Glycosylated hemoglobin						
mmol/L	64.0 ± 14.2	61.4 ± 12.2	60.7 ± 11.1	57.0 ± 17.8	1.1 [−1.98, 4.18]	.593
%	8 ± 1.3%	7.8 ± 1.1%	7.7 ± 1.0%	7.4 ± 1.6%		

Values are given as mean ± standard deviation or adjusted mean difference with 95% confidence interval.

Abbreviations: isCGM, intermittently scanned continuous glucose monitoring; DIY-rtCGM, do-it-yourself real-time continuous glucose monitoring.

a Confidence intervals and *P* values adjusted according to the Benjamini–Hochberg method.

### Sensor Use

Overall, sensor use in the DIY-rtCGM phase suggested a reduced percentage compared with isCGM but did not show any between-arm difference at the end of DIY-rtCGM compared with the end of isCGM.

### Participant-Reported Outcomes

There was an improvement in hypoglycemia fear score in both the worry subscale 0.24 [0.1, 0.38] and the total score 0.2 ([0.02, 0.38]; Table 3).

**Table 3.** Participant-Reported Outcomes (n = 52).

Scores	isCGM		DIY-rtCGM		Adjusted mean difference DIY-rtCGM vs isCGM [adjusted confidence intervals]^a^	*P* value
	Baseline	Eight weeks	Baseline	Eight weeks		
HFS (worry subscale)^b^ (range 0.1-2.5)	1.24 ± 0.70	1.18 ± 0.64	1.30 ± 0.64	0.97 ± 0.45	0.24 [0.1, 0.38]	**.02** ^g^
HFS (behavior subscale; range 0.2-3.7)	1.15 ± 0.55	1.13 ± 0.53	1.22 ± 0.48	0.99 ± 0.41	0.14 [−0.03, 0.31]	.157
HFS (total subscale; range 0.2-2.5)	1.21 ± 0.58	1.17 ± 0.54	1.26 ± 0.52	0.97 ± 0.37	0.20 [0.02, 0.38]	**.02**
PAID^c^ (range 3.75-68.75)	24.8 ± 14.5	23.6 ± 16.0	24.8 ± 15.3	20.3 ± 15.5	2.8 [−0.74, 6.35]	.157
DIDP (range 2.14-5.43)	3.18 ± 0.5	3.11 ± 0.4	3.18 ± 0.54	3.32 ± 0.51	−0.20 [−0.42 to −0.004]	**.045**
PSQI global^d^ (range 1-13)	7.9 ± 2.7	7.3 ± 2.8	7.5 ± 2.9	7.2 ± 2.9	0.34 [−0.49, 1.17]	.593
Gold score (range 1-5)	2.3 ± 1.0	2.4 ± 1.1	2.4 ± 1.3	2.2 ± 1.0	0.25 [−0.06, 0.56]	.175
DTSQ status (range 13-36)	24.8 ± 5.1	26.0 ± 5.8	26.3 ± 5.3	28.7 ± 5.8	2.7 [1.1, 4.3]	**.04**
DTSQ change^e^ (range −7-20)		8.3 ± 6.1		5.6 ± 8.5	2.7 [−0.51, 5.9]	.175
GMSS (range 1.9-3.6)	2.84 ± 0.3	2.86 ± 0.3	2.99 ± 0.5	2.96 ± 0.6	−0.04 [−0.14, 0.06]	.593
DDS-P^f^ (range 1-4.52)	1.90 ± 0.88	1.74 ± 0.7	1.73 ± 0.59	1.74 ± 0.49	−0.04 [−0.56, 0.48]	.739

Abbreviations: isCGM, intermittently scanned continuous glucose monitoring; DIY-rtCGM, do-it-yourself real-time continuous glucose monitoring; HFS, Hypoglycemia Fear Survey; PAID, Problem Area in Diabetes; DIDP, DAWN2 Impact of Diabetes profile; PSQI, Pittsburgh Sleep Quality Index; DTSQ, Diabetes Treatment Satisfaction Questionnaire; GMSS, Glucose Management Satisfaction Survey; DDS, Diabetes Distress Survey.

a Confidence intervals and *P* values adjusted according to Benjamini–Hochberg method.

b Values are mean items scores ± standard deviation of item scores.

c For PAID, DTSQ status where sum scores are used by convention. Two participants were below the age cutoff for PAID and therefore used PAID-T.

d Pittsburgh Sleep Quality Index, a score >5 indicates poor sleep quality.

e Compared using a paired *t* test.

f Data available for 27 participants.

g p<0.05 statistically significant.

Treatment satisfaction was better for DIY-rtCGM compared with isCGM, DTSQ status score 26.0 ± 5.8 versus 28.7 ± 5.8; mean difference 2.7 (95% confidence interval: [0.4%, 5.0%]); *P* = .04; mean difference in the DTSQ change score was also 2.7 (95% CI: [−0.51 to 5.9]; Table 3). Furthermore, there was no evidence of a difference between groups in monitoring device satisfaction using the GMSS (Table 3).

### Diabetes-Related Distress

No difference in overall diabetes-related distress was found between the two groups based on the PAID score, or in terms of severe diabetes distress (Table 4).

**Table 4.** Distribution of Severe Diabetes Distress for All Participants Across All Study Phases (n = 52).

	DIY-rtCGM Severe distress	DIY-rtCGM Less than severe distress
isCGM Severe distress	5	6
isCGM Less than severe distress	1	40

Severe distress is indicated by a Problem Area in Diabetes (PAID) score ≥40, whereas less than severe distress is indicated by a PAID score <40.

Abbreviations: DIY-rtCGM, do-it-yourself real-time continuous glucose monitoring; isCGM, intermittently scanned continuous glucose monitoring.

### Safety Outcomes: Adverse Events

Three participants (5.8%) were hospitalized during the study. No hospitalisations were attributable to either intervention. One of these admissions was due to COVID-19 infection during the DIY-rtCGM phase, whereas the other two were during the isCGM phase, for eye surgery (vitrectomy) and pneumonia (one case each). There were no admissions for diabetic ketoacidosis.

There were 25 adverse event episodes (as defined by the study protocol)^14^ in 18 participants (35%), with no severe adverse events or severe adverse device effects reported. Seven participants (39%) reported skin reactions with a total of nine episodes, one of which required oral antibiotics and the others required topical treatment with emollients or steroids alone. Six participants (32%) reported issues with DIY-rtCGM connectivity, resulting in four (22.2%) replacing DIY-rtCGM, one of whom replaced both their phone and DIY-rtCGM in an attempt to resolve the connectivity issues.

Two participants (8%) reported adverse events that were likely related to the manner in which DIY-rtCGM functions. These included an overtreated mild hypoglycemia episode, resulting in hyperglycemia due to issues with the lag or slow rise of sensor glucose, and one episode of moderate hypoglycemia not recognized due to a difference in readings (later found to be due to missed calibration by patient self-report. No episodes of severe hypoglycemia or hyperglycemia were found to be related to missed sensor glucose data in the process of event analysis. Two participants reported issues with isCGM (including the reader not charging and difficult sensor insertion).

## Discussion

This randomized controlled crossover trial provides much-needed data on clinical efficacy and participant-related outcomes for DIY-rtCGM use in adults. The TIR primary outcome and all other secondary glycemic metrics did not differ significantly between isCGM and DIY-rtCGM. At the time of study initiation, second-generation isCGM was not available in New Zealand. Even with the availability of second-generation isCGM with limited alarms,^7^ DIY-rtCGM potentially offers advantages over isCGM as a form of rtCGM that offers smartwatch accessibility (a feature that is still evolving in isCGM). Although objective measures were not significantly different between DIY-rtCGM and isCGM, a number of participant-reported outcomes were positive in favor of DIY-rtCGM. These included variables that indicated less fear of hypoglycemia and subtle improvements in treatment satisfaction in adults with type 1 diabetes using DIY-rtCGM. Sleep quality, measured using the Pittsburgh Sleep Quality Index, did not differ between the two monitoring devices.

Our finding of a lack of differences in TIR and time below range for DIY-rtCGM versus isCGM differs from previous studies comparing rtCGM with first-generation isCGM. These reported a 5.7% improvement in TIR for DIY-rtCGM versus isCGM in children aged <13 years,^12^ and a TIR improvement of up to 6.9% for commercial rtCGM compared with isCGM in adults in several trials.^8,10,11^ The absence of any positive effect of DIY-rtCGM on glycemic variables in this study may be partially explained by a range of participant-related and device-related factors. We found that overall sensor use in the DIY-rtCGM arm was low at appoximately 74%. This is much less than the proportion of sensor use in past clinical trials of rtCGM in adults.^16,22-24^ This suggests the importance of clinically relevant outcomes such as high sensor wear time, and potentially highlights the inherent technical and instability challenges of open-source DIY-rtCGM solutions. Technical challenges with DIY-rtCGM have been described previously.^12,13^ The results of our study suggest that, for the above reasons, DIY-rtCGM is unlikely to be as effective as commercial rtCGM for enhancing glycemic control in adults.

In contrast to the neutral result for glycemic improvements, participant-reported psychosocial outcomes were generally improved during use of DIY-rtCGM compared with isCGM. The improvements seen in the hypoglycemia fear score and diabetes treatment satisfaction of psychoscial outcomes were from questionnaires commonly evaluated in major longitudinal studies for both type 1 and type 2 diabetes internationally.^25,26^ These participant-reported data help to capture aspects of the diabetes experience that glycemic parameters alone do not,^25,27^ and add depth to outcome reporting.^27^ In terms of treatment satisfaction, the FUTURE study found that treatment satisfaction was better with rtCGM compared with isCGM.^28^ Better satisfaction with diabetes treatment has been associated with improvements in long-term glycemic control and complications.^29^ Most importantly, although a subtle improvement is seen in this study, better treatment satisfaction may result in better long-term persistence with treatment/monitoring. Overall, this is in keeping with the desire for new devices to reduce both the emotional and physical burden of type 1 diabetes. Overall, this is in keeping with the desire for new devices to reduce both the emotional and physical burden of type 1 diabetes.^30^

The improvements seen in psychosocial measures in our study highlight the fact that, in spite of technical challenges, DIY-rtCGM may still provide benefits to people with diabetes. These findings are consistent with the exisiting comparative rtCGM/isCGM literature.^8,10,12^ The use of a smartwatch was also a novel feature of this study. Previous qualitative work in children showed that this feature of DIY-rtCGM is well accepted and provides a discrete way to monitor glucose levels, and perhaps contributed to positive participant experiences. Watch technology may make sensor glucose information more accessible and more discrete, and may facilitate greater rtCGM data integration.

Challenges to continued use of DIY-rtCGM included technical issues such as loss of connectivity and reduced percent sensor usage. The impact of these challenges may be reflected in the slight reduction in diabetes-related quality of life scores on the DIDP because the sense of frustration caused by these technical challenges could have a negative impact on quality of life. In terms of connectivity, using the DIY-rtCGM system requires three separate digital interfaces, and the failure of any one of these can result in no glucose data being available to the user. Severe loss of connectivity, resulting in the replacement of devices was uncommon in our study, but impaired connectivity issues are well described in other studies.^12,13^ As outlined above, the pattern of reduced proportion of sensor use resulted in less glucose information available to effect changes in therapy during DIY-rtCGM. This in turn may be related to the need for calibration and the reduced overall battery life of the DIY-rtCGM system’s Bluetooth bridge, which is 10 days compared with 14 days for the isCGM sensor. Moreover, the overall mean absolute relative difference of DIY-rtCGM has not been measured to date, leading to both calibration and capillary glucose testing being recommended as stated. Furthermore, in terms of overall safety, use of the DIY-rtCGM device did not differ from isCGM. In terms of safety issues related to smartwatch integration, the smartwatch mainly acted as a medium to improve access to glucose levels taken from the phone receiver. Therefore the main potential risk of smartwatch integration was alarm fatigue, with the potential for alarms from both the phone (primary data collector) and watch. The overall frequency of cutaneous adverse events was similar to that reported in a systematic review.^31^

### Strengths and Limitations

The strengths of this study include the robust randomized crossover design. All participants were experienced isCGM users, which facilitated a stepwise comparison between the two technologies. Our data also provide first evidence regarding both the safety and acceptability of DIY-rtCGM in adults with type 1 diabetes, many of whom have used these devices in the absence of published evidence. Our study sample contained a high proportion of European participants, suggesting that the results may not be generalizable to ethnically diverse populations, and may reflect ongoing inequities in access to diabetes technology.^32^ Our findings also occurred in the context of a randomized clinical trial, where end user support was provided, including troubleshooting, which may be difficult to sustain in the context of routine clinical care. Both arms received this contact equally. At the time of study commencement, FreeStyle Libre (first-generation isCGM) was the only isCGM available in New Zealand (and in many locations worldwide); since then, second-generation devices with limited alarms (Free Style Libre 2) are now increasingly used, with third-generation rtCGM (Free Style Libre 3) devices having limited release worldwide as well. With all devices projected to be priced in a similar range, it is likely that need for DIY-rtCGM is likely to reduce over time. In the interim, DIY-rtCGM offers a potential solution to access rtCGM.

A number of diabetes associations have released advice around the use of DIY products, with Diabetes Australia and UK Diabetes bodies recommending that these devices cannot be prescribed or promoted but their use can be supported by clinician, with legal liability for correct use lying with the user.^33^ The use of licensed products, for example, the FreeStyle Libre sensor in this DIY-CGM, within the context of DIY solutions remains a contested legal area.^33^

## Conclusion

This is the first randomized controlled crossover trial in adults with type 1 diabetes to investigate glycemic control and participant-related outcomes in adult users of DIY-rtCGM. Although we found no improvements in glycemic outcomes, improvements in some participant-reported outcomes were seen, and no safety signals were seen. The results reflect a willingness to engage with DIY-rtCGM and provide insight into some of the challenges posed, including reduced sensor time percentage. The ongoing use of DIY-rtCGM requires a degree of technical understanding and a willingness to access open-source information for troubleshooting. Overall, DIY-rtCGM may be a valid potential alternative for such users who are unable to access rtCGM until such time as access improves, either through cheaper price or funded provision by health systems.
